# Effects of Immunonutrition in Head and Neck Cancer Patients Undergoing Cancer Treatment – A Systematic Review

**DOI:** 10.3389/fnut.2022.821924

**Published:** 2022-02-25

**Authors:** Sing Ean Tan, Nur Fadhlina Abdul Satar, Hazreen Abdul Majid

**Affiliations:** ^1^Centre for Population Health (CePH), Department of Social and Preventive Medicine, Faculty of Medicine, University of Malaya, Kuala Lumpur, Malaysia; ^2^Ministry of Health, Putrajaya, Malaysia; ^3^Department of Clinical Oncology, University of Malaya Medical Centre, Kuala Lumpur, Malaysia; ^4^Department of Nutrition, Faculty of Public Health, Airlangga University, Surabaya, Indonesia

**Keywords:** immunonutrition, glutamine, arginine, omega 3 fatty acid, radiotherapy, cancer treatment, head and neck (H&N) cancer

## Abstract

**Background and Aims:**

Malnutrition is prevalent among head and neck cancer (HNC) patients and leads to undesirable outcomes such as reduced treatment response and increased treatment-related side effects. This systematic review summarizes the recent evidence regarding the effect of immunonutrition in HNC patients undergoing radiotherapy and chemotherapy.

**Methods:**

A literature search was conducted of the CENTRAL, ProQuest, MEDLINE, EBSCOhost, Web of Science and CINAHL databases; and further supplemented with internet and manual searches. Studies published between January 2011 and May 2021 were identified, screened, retrieved, and data extraction was performed.

**Results:**

Twenty studies involving 1535 patients were included, 15 were randomized controlled trials (RCTs), three were retrospective study and two were comparative cohort studies. Five out of seven studies reported improvement or maintenance of nutrition status with continuous supplementation using immunonutrient-enriched formula. Three studies reported functional status as an outcome, with one study reporting significant improvement, one study reporting maintenance, and another study reporting no difference in the functional status of patients supplemented with immunonutrient-enriched formulas. Supplementation with glutamine did not reduce the overall incidence of mucositis but delayed the onset of oral mucositis and had significantly less incidence of severe oral mucositis.

**Conclusion:**

Supplementation with immunonutrient-enriched formulas in HNC patients during radiotherapy and chemotherapy may improve or maintain nutrition status. Supplementation with glutamine during HNC radiotherapy and chemotherapy may delay the onset of oral mucositis and reduce incidences of severe oral mucositis. Further investigations are required, focusing on the timing, dosage, and duration of immunonutrition.

**Systematic Review Registration:** PROSPERO, identifier CRD42021241817.

## Introduction

Head and neck cancer refers to neoplasms occurring in the head and neck region, including the pharynx, nasal, and oral cavity, metastasising to cervical neck nodes. The curative treatment of HNC includes concurrent chemoradiotherapy, radiotherapy alone, or postoperative radiotherapy.

Malnutrition in cancer patients is associated with weight loss, reduced immune competence, increased risk of infections, increased treatment toxicities, and greater mortality risk. The prevalence of malnutrition is very high in cancer patients undergoing treatment (1–3). Patients with primary cancers involving the gastrointestinal tract, head and neck, liver, and lung are exceptionally at high risk of malnutrition (1). In HNC, the prevalence of malnutrition is at an alarming 22–56% upon diagnosis (4–6). Malnutrition in cancer patients can be attributed to inadequate nutritional intake, likely due to primary anorexia or secondary causes (e.g., mucositis, xerostomia, intestinal obstruction, malabsorption, nausea, vomiting, pain, etc.). Additionally, metabolic derangements such as increased metabolism and catabolism further reduce cancer patients' nutrition status. For cancer patients undergoing cancer treatment, malnutrition increases the risk of treatment-related toxicities, resulting in treatment withdrawal and eventual reduction in treatment response.

Immunonutrition can be defined as modulation of either the immune system activity or modulation of the consequences of activation of the immune system by nutrients or specific food items fed in amounts above those typically encountered in the diet (7). Immunonutrients identified and studied are omega-3 fatty acids, glutamine, arginine, branched-chain amino acids, and nucleotides (8–10). Immunonutrition can be provided in the form of immunonutrient-enriched formula, single immunonutrient, or combination of immunonutrients. Immunonutrition was found to reduce the severity of treatment-related toxicities such as oral mucositis, diarrhea, oesophagitis, and weight loss (11, 12). However, the variability in the type, dose, and duration of immunonutrition led to inconsistent outcomes among available evidence.

This systematic review summarizes the recent evidence regarding the effect of immunonutrition in HNC patients undergoing radiotherapy and chemotherapy.

## Materials and Methods

This systematic review was designed according to the PICOS criteria outlined in Table 1 and reported according to the Preferred Reporting Items for Systematic Reviews and Meta-Analyses (PRISMA) 2020 Statement guidelines. The protocol of this systematic review was registered in the International Prospective Register of Systematic Reviews (PROSPERO) under the registration number: CRD42021241817.

**Table 1:** PICOS Criteria.

**Criteria**	**Description**
Participants	HNC patients undergoing radiotherapy and/or chemotherapy
Intervention/Exposure	Supplementation with immunonutrition -including arginine, glutamine, omega-3 fatty acids, nucleotides; isolated or combined; administered via oral supplementation or enteral route
Comparison	Any parallel group with similar clinical properties, receiving standard care, with or without nutrition supplementation
Outcomes	Nutrition status, functional status, treatment-related toxicities
Study Design	RCT, non-RCT (e.g., controlled clinical trial)

### Search Strategy

A literature search was conducted of six databases: Cochrane Central Register of Controlled Trials (CENTRAL) in The Cochrane Library, ProQuest, MEDLINE (Pubmed), EBSCOhost, Web of Science and CINAHL. The literature search was further supplemented with internet searches (e.g., Google Scholar) and a manual search of the reference lists of relevant studies and previously published systematic reviews. Studies published from January 2011 to May 2021 were included in the search. There were no language restrictions for the studies.

The search strategy included three groups of keywords and Medical Subject Headings (MeSH) terms that describe immunonutrition, head and neck cancer patients, and cancer treatment. Search terms of the same group, such as “immunonutrition,” “immune-enhancing nutrition,” “immune-modulating nutrition,” “glutamine,” “arginine,” omega 3 fatty acid,” “fish oil,” “nucleotides” were combined using Boolean operator OR. Search terms for the three different groups were then combined with the Boolean operator AND (refer to Supplementary Material).

### Eligibility Criteria

The inclusion criteria for studies to be considered for this review were (1) primary research involving adult (above 18 years) HNC patients undergoing radiotherapy and or chemotherapy either as primary treatment modality or post-operatively; (2) comparing immunonutrition (combination of immunonutrients or involve at least one immunonutrient – glutamine, arginine, omega 3 fatty acid) vs. standard nutrition (polymeric nutrition formula that is nutritionally complete), or placebo or no nutrition intervention; (3) reported nutrition status, functional status and treatment-related toxicities as outcomes.

Studies that did not meet the inclusion criteria were excluded: involving participants <18 years old, involving participants who did not undergo radiotherapy or chemotherapy, and involving nutrition supplementation via parenteral nutrition. Duplicate and irrelevant studies were also excluded in case reports, letters, reviews, animal or *in vitro* studies.

### Study Selection and Data Collection

The selection of articles involved three stages: (1) selection based on title, (2) abstract consideration, (3) assessing the full text. Two reviewers independently assessed the potentially relevant articles for eligibility. Disagreements are resolved through discussion until consensus is reached. A third reviewer was consulted in the event that no consensus was reached.

Database searches and reference lists were imported into EndNote™ 20, Clarivate Analytics (US) LLC. Data extraction was performed using a data extraction table that collects information such as bibliography information (title, author, publication year, journal, country/institution where the study was conducted), study design, study duration, study population (inclusion/exclusion criteria, sample size, type of cancer, type of treatment), intervention, comparison, outcomes, etc. Study investigators were contacted to clarify or obtain more information when necessary. Two reviewers independently extracted the data. Discrepancies are resolved through discussion until consensus is reached. A third reviewer was consulted in the event no consensus was reached.

### Outcomes

The primary outcome specified was nutrition status, which included: changes in weight and BMI, body composition, Subjective Global Assessment (SGA), and Nutritional Risk Index (NRI).

Secondary outcomes that were specified were functional status and treatment-related toxicities. Functional status is measured by handgrip strength or performance scores such as ECOG, Kondrup, or Karnofsky Performance Index. Incidences and severity of treatment-related toxicities are graded using National Cancer Institute Common Terminology Criteria for Adverse Events (CTCAE) v5.0.

### Quality and Risk of Bias Assessment

Two reviewers performed the quality and Risk of Bias assessment independently, using the Jadad Scale (13) and Cochrane Risk of Bias Tool (14) for randomized, controlled trials. Results were compared, and any discrepancies were resolved through discussion; a third reviewer was consulted on the occasion where consensus could not be reached. The methodological quality of controlled trials was scored according to three areas – randomisation, masking and accountability. The bias of the studies was rated as High, Low or Unclear; on five specified domains (Selection, Performance, Attrition, Reporting, and Other).

### Data Synthesis

Narrative synthesis of the information gathered in the data extraction form is structured around the type of intervention, target population characteristics, type of outcome, and intervention content. Summary of intervention effects were tabulated.

## Results

**Figure 1:**
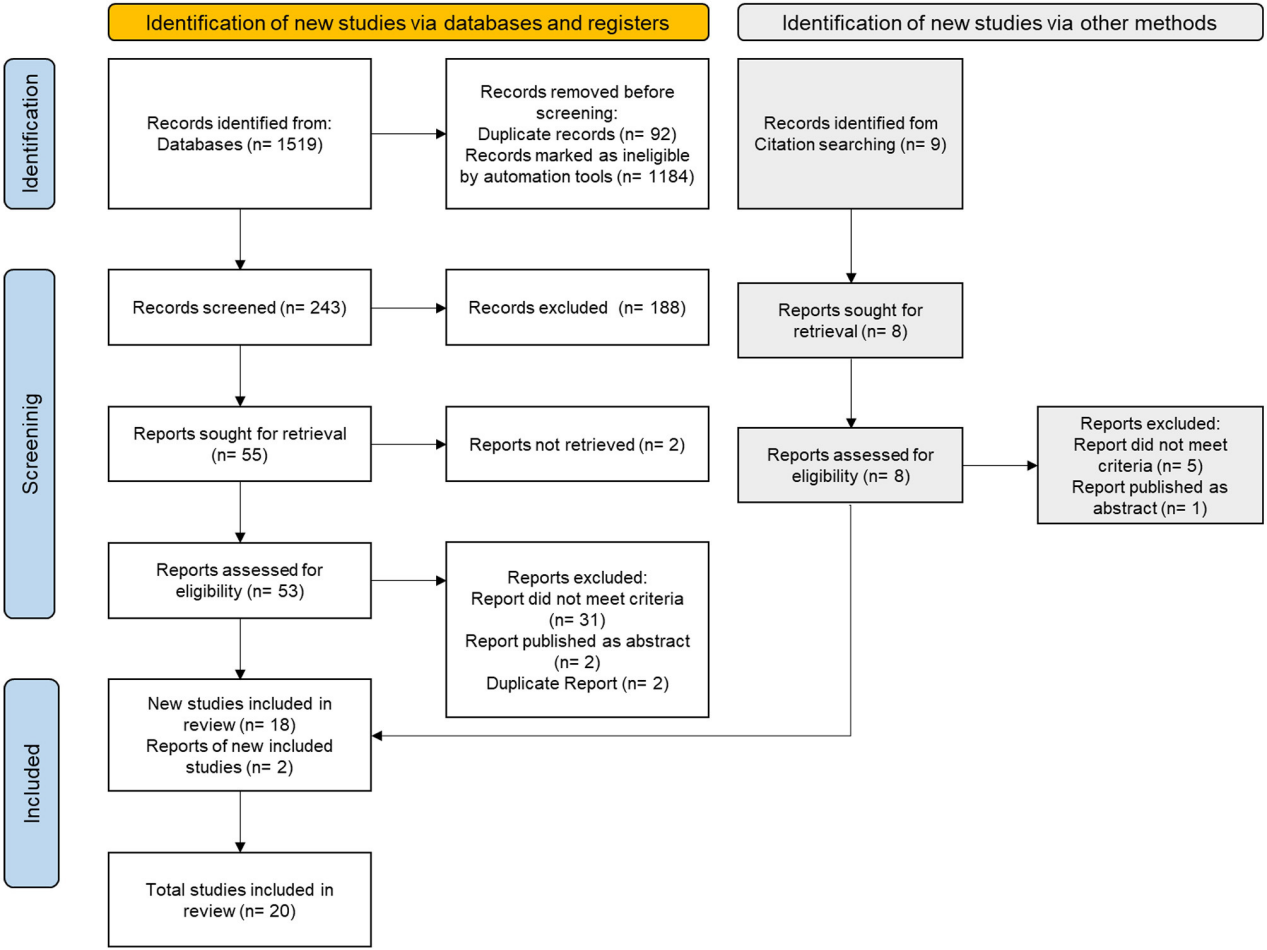
Flow diagram of study selection.

The literature search identified 1,519 articles. Nine other articles were identified through reference list and citation search. Duplicate articles and ineligible articles were removed via automation tools or manual identification. The remaining 243 articles were screened based on title and abstract. The full texts of 62 articles were then retrieved and assessed. Thirty-six studies were excluded because they did not meet the eligibility criteria, and two studies were excluded because of duplication. Three studies published as abstracts were excluded because retrieval of the full manuscript was unsuccessful as there was no reply from the authors (15–17). Finally, a total of 20 studies were included in this systematic review. The study selection process is outlined in Figure 1.

Characteristics of the studies included are summarized in Table 2. The sample size of the studies ranges between 26 and 262, with an accumulative total of 1,535 patients, of which 805 received immunonutrition while 730 received standard nutrition or placebo or no treatment. The studies are categorized according to the type of intervention, including supplementation using immunonutrient-enriched formula, or supplementation using a single immunonutrient or combination of immunonutrients. Ten studies involved supplementation using immunonutrient-enriched formula (18–27), while nine studies involved the supplementation of a single immunonutrient (glutamine) (28–36), and one study involved supplementation of immunonutrients (glutamine and arginine) with hydroxy-beta-methylbutarate (37). Majority of the studies involved only HNC on radiotherapy with or without chemotherapy, except for five studies that involved HNC and oesophageal cancer patients in their study population (19, 23, 25, 36, 38).

**Table 2:** Summary of studies included in the systematic review.

**ID**	**Study and country**	**Study design**	**Type of cancer, treatment**	***N* = (IG,CG)**	**Duration of supplementation and Intervention**
**Immunonutrient-enriched Formulas**					
1	Boisselier (18) 2020 France	Prospective, randomized, controlled, double-blind, multicenter	HNC, RTx/CTx	172 (86, 86)	Interval (5 days before each CTx cycle) IG: IN (Oral Impact – L-arginine, n-3 FAs, ribonucleic acids) CG: SN (isocaloric, isonitrogenous) 3 servings/day
2	Chitapanarux (19) 2019 Thailand	Prospective, randomized, controlled, not blinded, multicenter	HNC, oesophageal & cervical ca, RTx/CTx	88 (44, 44)	Continuous throughout treatment (5–7 weeks) IG: regular diet + IN (Neo-Mune - arginine, glutamine, fish oil) 2 servings/day or enteral IN before and after RTx CG: regular diet or enteral SN
3	Harada (20) 2019 Japan	Prospective, randomized, not blinded, single center	Oral SCC, RTx/CTx	50 (25, 25)	Continuous throughout treatment (6–7 weeks) IG: IN (Elental – elemental formula with glutamine), throughout treatment 1bottle/day CG: no treatment
4	Chitapanarux (21) 2016 Thailand	Prospective, randomized, not blinded, single center	HNC, RTx/CTx	40 (20, 20)	Continuous throughout treatment (7 weeks) IG: nutrition counseling + IN (Neo-mune – arginine, glutamine, MCT, fish oil) 2 servings/day before and after RTx CG: nutrition counseling only
5	Vasson (23) 2014 France	Prospective, randomized, controlled, double-blind, multicenter	HNC & oesophageal ca, RTx/CTx	28 (15, 13)	Continuous, 5 days before initiation of RTx until end of treatment (5–7 weeks) IG: enteral IN (Impact – arginine, EPA & DHA, ribonucleotides) CG: SN (Isosource – isocaloric, isonitrogenous, polymeric)
6	Roca-Rodriguez (24) 2014 Spain	Prospective, randomized, controlled, not blinded, single center	ENT ca, RTx	26 (13, 13)	Continuous, 14 days after initiation of RTx until 90 days post RTx IG: IN (Prosure – 3 servings/day, add on with standard formula CG: SN (Isosource – standard, polymeric)
7	Fietkau (25) 2013 Germany	Prospective, randomized, controlled, double-blind, multicenter	HNC & oesophageal ca, RTx/CTx	69 (38, 31)	Continuous throughout treatment (up till 14 weeks) IG: SN + IN (Supportan – high fat, high protein, fish oil) 500ml via PEG feeding CG: SN (Fresubin Energy Fibre) via PEG feeding allowed orally
8	Yeh (26) 2013 Taiwan	Prospective, randomized, controlled, not blinded, single center	HNC, RTx/CTx	68 (31, 37)	Continuous throughout treatment until 1 month post treatment (3 months) IG: IN (Ethanwell – protein & energy-densed, n-3 FAs, glutamine, selenium, CoQ10; Ethanzyme - probiotics) CG: SN (Isocal)
9^*^	Chao (27) 2020 Taiwan	Retrospective, single center	HNC & oesophageal ca, RTx/CTx	88 (44, 44)	Continuous throughout treatment (> 7 days supplementation) IG: IN (Oral Impact – L-arginine, n-3 FAs, ribonucleic acids) CG: SN (isocaloric, isonitrogenous) 3 servings/day
10^*^	Yuce Sari (22) 2016 Turkey	Prospective, Not randomized, not blinded, single center	HNC, RTx/CTx	29 (15, 14)	Continuous throughout treatment (5–7 weeks) IG: IN (Abound – glutamine, arginine) throughout treatment CG: no treatment
**Immunonutrients**					
11	Huang (29) 2019 Taiwan	Prospective, randomized, controlled, double-blind, single center	HNC, RTx/CTx	59 (30, 29)	Continuous, 1 week before initiation of RTx until 2 weeks post RTx (8 weeks) IG: L-glutamine 10g + maltodextrin 5g CG: placebo – maltodextrin 15g 3x/day
12	Pathak (28) 2019 India	Prospective, randomized, controlled, not blinded, single center	Oropharynx & larynx ca, RTx/CTx	56 (28, 28)	Continuous, 5 days/week during treatment (7 weeks) IG: glutamine 10 g 2 h before RTx CG: no treatment
13	Lopez-Vaquero (32) 2017 Spain	Prospective, randomized, controlled, double-blind, single center	HNC, RTx/CTx	49 (25, 24)	Continuous throughout RTx (6 weeks) IG: glutamine 10 g CG: maltodextrin 10 g 3x/day
14	Pattanayak (33) 2016 India	Prospective, randomized, controlled, not blinded, single center	HNC, RTx/CTx	162 (81, 81)	Continuous throughout RTx (7 weeks) IG: glutamine 15 g 2x/day CG: no treatment
15	Tsujimoto (34) 2015 Japan	Prospective, randomized, controlled, double-blind, single center	HNC, RTx/CTx	40 (20, 20)	Continuous throughout RTx (6–7 weeks) IG: glutamine 10 g CG: placebo 10 g 3x/day
16	Imai (37) 2014 Japan	Prospective, randomized, controlled, not blinded, single center	HNC, RTx/CTx	34 (16, 18)	Continuous throughout RTx until 1 week post RTx (7–8 weeks) IG: HMB+Arg/Gln (Abound – beta-hydroxy-beta-methylbutarate, L-arginine, L-glutamine) 2x/day CG: no intervention active prophylactic enteral tube feeding
17	Chattopadhyay (35) 2014 India	Prospective, randomized, not blinded, case control, single center	HNC, RTx/CTx	70 (35, 35)	Continuous, 5 days/week during treatment IG: glutamine 10 g 2 h before RTx CG: no treatment
18^*^	Akmansu (30) 2018 Turkey	Retrospective, single center	HNC, RTx/CTx	28 (18,10)	Continuous throughout treatment (5–7 weeks) IG: L-glutamine 10 g 3x/day CG: no treatment
19^*^	Pachon Ibanez (31) 2018 Spain	Prospective, non-randomized, comparative, cohort, single center	HNC, RTx/CTx	262 (131,131)	Continuous throughout RTx (7 weeks) IG: glutamine 10g 3x/day CG: no treatment
20^*^	Vidal-Casariego (36) 2013 Spain	Retrospective, non-randomized, cohort	HNC, lung, oesophageal ca RTx to head and neck and chest area	117 (32, 58, 27)	Up to 6 weeks Glutamine 30 g/day IG A: Early treatment - received glutamine before initiating and during RTx IG B: Delayed treatment - received glutamine when RTx had already begun CG: Not treated - did not receive any glutamine during RTx

*Arg, arginine; CG, control group; CTx, chemotherapy; DHA, docosahexaenoic acid; EPA, eicosapentanoiec acid; FA, fatty acid; Gln, glutamine; HMB, beta-hydroxy beta-methylbutyric acid; IG, intervention group; IN, immunonutrition formula; RTx, radiotherapy, SN, standard nutrition formula*

* *Non-RCT studies*.

Most of the studies involved immunonutrition as oral nutrition supplements and are only administered via a feeding tube when the subjects were unable to tolerate it orally. For the three studies that involved oesophageal cancer patients, the immunonutrient-enriched formula was administered via a feeding tube upon initiation of intervention (19, 23, 25).

### Quality and Risk of Bias Assessment

**Figure 2:**
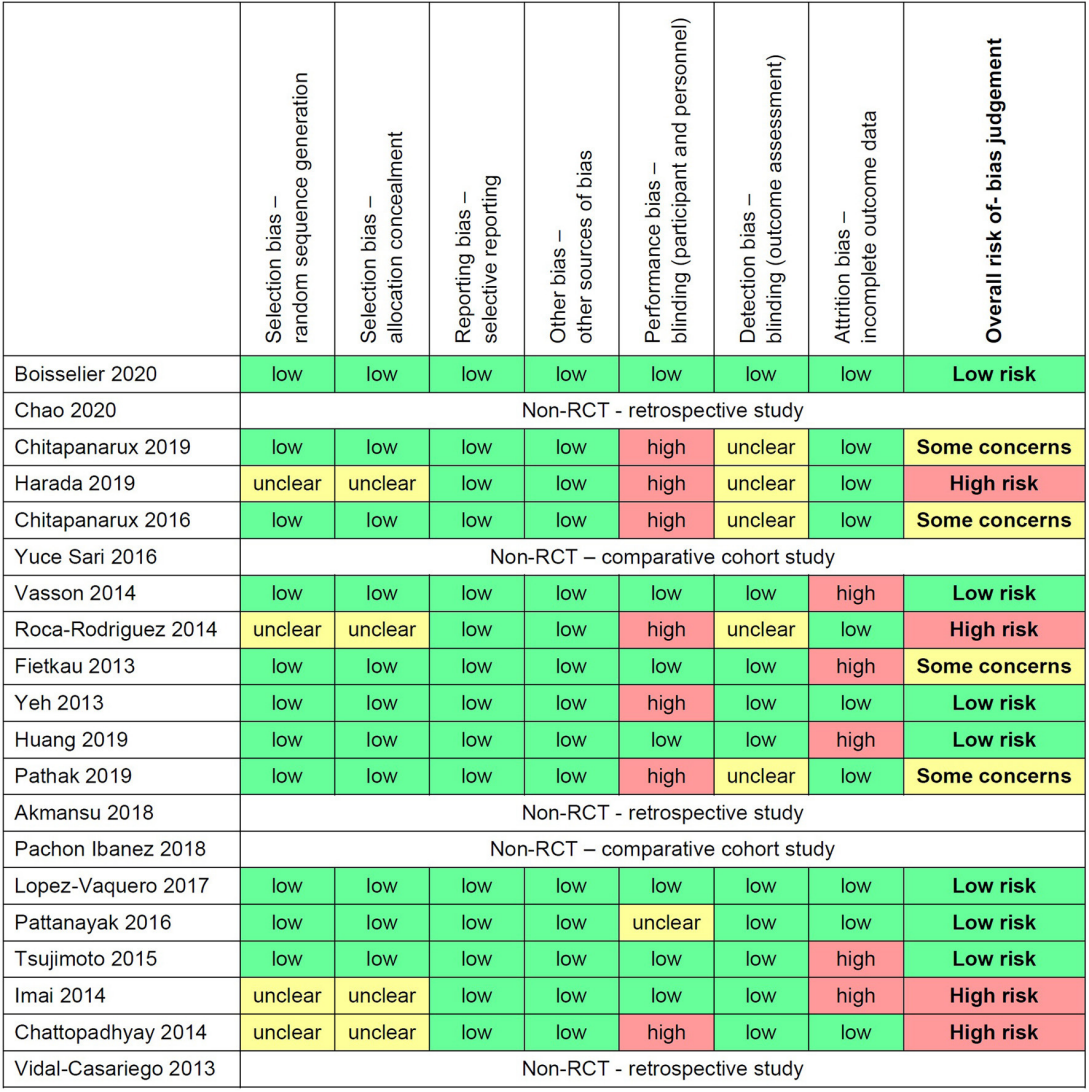
Summary of risk of bias assessment using the Cochrane Risk of Bias Tool for Randomized Control Trails.

The risk of bias of the included studies was evaluated using the Cochrane Risk of Bias Tool for Randomized Control Trials and summarized in Figure 2. Out of the 15 studies that were evaluated, seven were classified under low risk of bias, four were classified as high risk of bias, and four were judged to have raised some concerns of risk of bias. The most common source of bias was performance bias (i.e., blinding of participants and personnel). Seven studies were non-blinded as they were either open-label studies or the control group did not receive any treatment. For selection bias, four studies did not describe in detail the randomisation process or participant allocation. Therefore, the risk of bias was unclear. In terms of detection bias, five studies did not describe if the outcome assessors were blinded to the intervention allocation or not. Hence the risk of bias was unclear. Finally, for attrition bias, five studies were classified as high risk as there was more than 10% dropout or loss of sample.

The quality of the clinical trials was also assessed using the Jadad scale and summarized in Table 3. Twelve studies were of high quality (score between 3 and 5), whereas three were low quality (score between 0 and 2).

**Table 3:** Summary of methodological quality assessment using the Jadad Score.

	**Study described as randomized**	**Method to generate the sequence of randomisation described and appropriate**	**Study described as double-blind**	**Method of double-blinding described and appropriate**	**Description of withdrawals and dropouts**	**Overall score**
Boisselier 2020	1	1	1	1	1	5
Chao 2020	Non-RCT - retrospective study					
Chitapanarux 2019	1	1	0	0	1	3
Harada 2019	1	0	0	0	1	2
Chitapanarux 2016	1	1	0	0	1	3
Yuce Sari 2016	Non-RCT - comparative cohort study					
Vasson 2014	1	1	1	1	1	5
Roca-Rodriguez 2014	1	1	0	0	1	3
Fietkau 2013	1	1	1	1	1	5
Yeh 2013	1	1	0	0	1	3
Huang 2019	1	1	1	1	1	5
Pathak 2019	1	1	0	0	1	3
Akmansu 2018	Non-RCT - retrospective study					
Pachon Ibanez 2018	Non-RCT – comparative cohort study					
Lopez-Vaquero 2017	1	1	1	1	1	5
Pattanayak 2016	1	1	0	0	1	3
Tsujimoto 2015	1	0	1	1	1	4
Imai 2014	1	0	0	0	1	2
Chattopadhyay 2014	1	0	0	0	1	2
Vidal-Casariego 2013	Non-RCT - retrospective study					

### Immunonutrient-Enriched Nutrition Formula

Ten studies evaluated the effects of immunonutrient-enriched nutrition formulas. Of these, three involved nutrition formula enriched with arginine and omega-3 fatty acids (18, 23, 27); two involved nutrition formula enriched with omega-3 fatty acids (24, 25); two involved nutrition formula enriched with arginine, glutamine and omega-3 fatty acids (19, 21); one involved nutrition formula enriched with glutamine and arginine (22); one involved elemental nutrition formula containing glutamine (20); and one involved nutrition formula enriched with glutamine and omega-3 fatty acids (26). In terms of methodological quality, seven out of 10 of the studies were of good quality.

Most studies involved continuous supplementation during and throughout the radiotherapy and chemotherapy treatment, except for Boisselier et al. that provided immunonutrient-enriched formula in intervals (5 days before each chemotherapy cycle) (18); Roca-Rodriguez et al. that started immunonutrient-enriched formula 14 days after initiation of radiotherapy and continued up to 90 days post-radiotherapy treatment (24); and Yeh et al. that continued the immunonutrient-enriched formula until one-month post-radiotherapy treatment (26).

In terms of nutritional status, five studies found significant improvements in the nutrition status for patients in the intervention group (21, 23, 25–27). On the other hand, two studies observed no difference between the intervention and control groups (20, 24). For treatment-related toxicities, three studies reported reduced incidence and severity of oral mucositis in the intervention group (20–22), while three other studies found no difference between groups (18, 23, 24). Hematological toxicities were reported to be higher in the control group by two studies (19, 21), while one study reported no difference between groups (24). Only three studies measured functional status as an outcome. Fietkau et al. reported significantly improved functional status (improved Karnofsky Performance Index score) in the intervention group (25), Vasson et al. reported maintenance of functional status in the intervention group compared to the control group who had deterioration of functional status (increased WHO Performance Status score and decreased Karnofsky Index score) (23), while Roca-Rodriguez et al. reported no difference in the functional status between control and the intervention group (24). The results of the studies are summarized in Tables 4, 5.

**Table 4:** Summary of results.

**ID**	**Study**	**Results**	**Improvement or Maintenance of Nutrition Status**	**Improvement or Maintenance of Functional Status**	**Incidence and Severity of Treatment-related Toxicities**
**Immunonutrient-enriched Formulas**					
1	Boisselier (18) 2020	Nutrition Status: - Functional Status: - Treatment-related toxicities: no difference in severe oral mucositis rate – IG 33.7%, CG 34.9% Overall survival and progression free survival 3 years post treatment improved in IG 77% & 70%, CG 68% & 59%	NA	NA	↔
2	Chitapanarux (19) 2019	Nutrition Status: - Functional Status: - Treatment-related toxicities: Higher incidences of hematological toxicities in CG than IG (*p* = 0.03) Higher percentage of grade 3–4 non-hematological toxicities in CG than IG, but not significant (*p* = 0.2)	NA	NA	-
3	Harada (20) 2019	Nutrition status: No significant difference in body weight between groups Functional Status: - Treatment-related toxicities: Significantly lower grade of mucositis in IG (*p* = 0.0006) Significantly lower rates of severe mucositis during chemoradiation in IG 4.76% than CG 77.8% (*p* < 0.0001)	↔	NA	-
4	Chitapanarux (21) 2016	Nutritional status: Significant weight loss in CG 56.3–47.0kg (*p* < 0.001), maintained in IG 60.0–53.0 kg (*p* = 0.109) Functional Status: - Treatment-related toxicities: Non-hematological toxicities - oral mucositis 20% in CG, 5% in IG; radiation dermatitis 5% in CG, 0% in IG Severe hematological toxicities - significantly higher incidences in CG than IG (*p* = 0.035) Alb and pre-alb reduced in both groups, but median alb in IG significantly higher in IG (*p* = 0.028) at end of treatment	+	NA	-
5	Vasson (23) 2014	Nutritional status: Weight – significantly increased in IG (+1.8 ± 2.7kg) BMI - significantly increased in IG (+10.7 ± 0.9kg/m^2^) lean body mass - significantly increased in IG (+2.1 ± 3.2kg) Functional status: Deterioration of functional capacities in CG – increased WHO PS score and decreased Karnofsky Index Upper arm muscular strength maintained in both IG & CG – no significant difference Treatment-related toxicities: no significant difference for mucositis QOL: EORTC-QLQ C30, QOL-H&N35 – no significant difference between groups	+	+	↔
6	Roca-Rodriguez (24) 2014	Nutrition status: BMI decreased during treatment, then recovered post treatment, no significant difference between groups Functional status: No significant difference between groups for Karnofsky Performance Index during treatment and post treatment Treatment-related toxicities: No significant difference between groups for haematologic/mucosal/skin toxicity	↔	↔	↔
7	Fietkau (25) 2013	Nutritional status: improved NRS score, body cell mass, body weight, BMI, MAC in IG, but not significant Kondrup score – significant improvement in IG compared with CG (*p* = 0.0165) SGA score – IG 28.6% improvement and 71.4% no change; CG 3.3% improvement, 86.7% no change, 10% deteriorate (*p* = 0.0065) Functional status: Significant improvement of Karnofsky Performance Index in IG (p=0.04), less decreased in hand grip strength in IG but not statistically significant Treatment-related toxicities: - QOL: EORTC-QLQ C30 – no significant difference between groups	+	+	NA
8	Yeh (26) 2013	Nutritional status: Weight – IG+BMI <19 weight gain 9.0%; CG+BMI <19 weight loss 7.3% (p <0.05) maintenance and improved alb & pre-alb levels in IG where BMI <19 Functional Status: - Treatment-related toxicities: -	+	NA	NA
9^*^	Chao (27) 2020	Nutritional status: Significant increase in weight (0.97 ± 2.7 kg) in IG, but significant decrease in CG (-0.90 ± 1.49 kg) Significant increase in BMI (0.35 ± 1.02 kg/m^2^) in IG, but significant decrease in CG (−0.33 ± 0.54 kg/m^2^) Significant increase in MAMC (0.26 ± 0.72 cm) in IG, but significant decrease in CG (−0.27 ± 0.70 cm) Better PG-SGA score for IG compared to CG (*p* = 0.048) NRI significantly increased in IG (0.67 ± 1.85), but decreased in CG Functional Status: - Treatment-related toxicities: -	+	NA	NA
10^*^	Yuce Sari (22) 2016	Nutrition Status: - Functional Status: - Treatment-related toxicities: Significantly higher stomatitis scores, oral mucositis scores, oral pain scores, dysphagia scores in CG compared to IG QOL: No significant difference between groups for global health score, functional scale. Significant lower social function score, and higher symptom scale score in CG	NA	NA	-
**Immunonutrients**					
11	Huang (29) 2019	Nutrition status: Decrease of BMI strongly correlated with severe oral mucositis Functional Status: - Treatment-related toxicities: Significantly lower incidence of severe oral mucositis in IG (*p* = 0.045) Significant difference between groups for mean maximum mucositis grade, IG 1.6 ± 0.6 compared to CG 2.1 ± 0.8 (*p* = 0.009) No difference between groups for development of dermatitis (*p* = 0.221)	NA	NA	-
12	Pathak (28) 2019	Nutrition status: Significant weight loss >3 kg, CG 100% compared to IG 71% Functional Status: - Treatment-related toxicities: Significantly later time to onset and less severity of oral mucositis and dysphagia in IG compared to CG	+	NA	-
13	Lopez-Vaquero (32) 2017	Nutrition status: No significant difference between groups for weight loss Functional Status: - Treatment-related toxicities: Incidence and severity of oral mucositis – no significant difference between groups Significantly lower incidence and severity of dermatitis in IG compared to CG (*p* = 0.038 and *p* = 0.032)	↔	NA	- ^∧^
14	Pattanayak (33) 2016	Nutrition Status: - Functional Status: - Treatment-related toxicities: Onset of oral mucositis – 55% of CG at week 3, 55% of IG at week 5 Severity of mucositis – 92% CG developed grade 3 mucositis, none of IG developed grade 3 mucositis Less incidence of pain/dysphagia/nausea/cough in IG	NA	NA	-
15	Tsujimoto (34) 2015	Nutrition status: NRS score significantly lower in IG (p < 0.05) Mean % weight change – IG 3.6%, control 6.0% Functional Status: - Treatment-related toxicities: Maximal mucositis grade and mean mucositis grade significantly lower in IG 2.9 ± 0.3, CG 3.3 ± 0.4 (*p* = 0.005) Mean time to mucositis onset and mucositis duration no significant difference between groups (*p* = 0.663 and *p* = 0.6717)	+	NA	-
16	Imai (37) 2014	Nutrition Status: - Functional Status: - Treatment-related toxicities: Incidence of >grade 2 dermatitis significantly lower in IG 62.6% compared to CG 94.4% (*p* = 0.029) Duration of dermatitis significantly shorter in IG 44.8% compared to 56.7% (*p* = 0.009)	NA	NA	-
17	Chattopadhyay (35) 2014	Nutrition Status: - Functional Status: - Treatment-related toxicities: No significant difference between groups in development of oral mucositis Significantly lower incidence of severe mucositis (grade 3 & 4) in IG (*p* = 0.02 and *p* = 0.04) Significantly less mean duration of severe oral mucositis in IG 6.6 days compared to CG 9.2 days (*p* < 0.001) Significantly earlier onset of oral mucositis in CG (*p* < 0.001)	NA	NA	-
18^*^	Akmansu (30) 2018	Nutrition status: No significant difference between groups for weight changes Functional Status: - Treatment-related toxicities: No significant difference between groups for incidence of oral mucositis (42.1% and 44.4%), but significantly lower incidence for severe mucositis >grade 3 in IG 5.3% compared to CG 55.6% (*p* = 0.008) CG significantly earlier onset of mucositis at 14^th^ day compared to IG at 18^th^ day	↔	NA	-
19^*^	Pachon Ibanez (31) 2018	Nutrition Status: - Functional Status: - Treatment-related toxicities: Incidence of oral mucositis lower in IG 50.4% compared to CG 59.5%, but not significant (*p* = 0.55) Incidence of odynophagia lower in IG 55.7% compared to CG 77.9% (*p* = 0.0001)	NA	NA	- ^∧^
20^*^	Vidal-Casariego (36) 2013	Nutrition status: Occurrence of weight loss – IG A 6.6%, IG B 9.2%, CG 13.1%, significant difference between groups (*p* = 0.008) Significantly less weight loss in IG A 5.6 kg, IG B 11.3 kg, CG 13.4 kg (0.009) Functional Status: - Treatment-related toxicities: Development of oral mucositis – IG A 75%, IG B 94.7%, CG: 100%, significant difference between IG A and CG Severity of oral mucositis lower in IG A Risk of mucositis for patients receiving glutamine −14%, 95% CI	+	NA	-

+ *Indicates significant improvement or maintenance of nutritional status or functional status in the intervention group compared to control group (p < 0.05)*.

↔ *Indicates non-significant results (p > 0.05)*.

- *Indicates significant lower incidence or severity of treatment-related toxicities in the intervention group compared to control group (p < 0.05)*.

*NA outcome not being studied or reported^∧^ no significant difference for severity and incidence of oral mucositis, but significantly lower incidence and severity of dermatitis in one study and lower incidence of odynophagia in another*.

* *non-RCT studies*.

**Table 5:** Summary of results according to types of formulas or immunonutrients.

	**Number of studies**	**Outcomes**	**Positive results (*p* < 0.05)**	**Non-significant results (*p* > 0.05)**
**Immunonutrient-enriched Formulas**				
Nutrition formula with omega-3 fatty acids	2	Nutritional status	1	1
		Functional status	1	1
		Treatment-related toxicities		1
Nutrition formula with omega-3 fatty acids + arginine and/or glutamine	6	Nutritional status	4^#^	
		Functional status	1	
		Treatment-related toxicities	2	2
Nutrition formula with arginine and glutamine	2	Nutritional status		1
		Treatment-related toxicities	1^#^	
**Immunonutrients**				
Glutamine	9	Nutritional status	3^#^	2^#^
		Treatment-related toxicities (oral mucositis)	7^#^	2^*^^#^
Glutamine + Arginine with HMB	1	Treatment-related toxicities (dermatitis)	1	

*Nutrition status is measured by changes in weight and BMI, body composition, Subjective Global Assessment (SGA), and Nutritional Risk Index (NRI)*.

*-Functional status is measured by handgrip strength or performance scores such as ECOG score, Kondrup score or Karnofsky Performance Index*.

*-Positive results depicts improvement or maintenance of nutritional status and functional status, or lower incidence or severity of treatment-related toxicities*.

* *No significant difference for severity and incidence of oral mucositis, but significantly lower incidence and severity of dermatitis in one study and lower incidence of odynophagia in another*.

# *Contains references that are non-RCT studies*.

### Glutamine

Nine studies evaluated the effects of supplementation with single immunonutrient (glutamine) vs. placebo or no treatment (28–36). All studies involved continuous supplementation with 10 to 30 grams of glutamine per day and supplementation period ranging between five to eight weeks. One study involved continuous supplementation until one-week post-treatment with a combination of immunonutrients (arginine and glutamine) with HMB (37). However, only five studies were classified as having good methodological quality, with a Jadad score between 3 and 5. Two studies were of poor methodological quality, and three other studies were non-randomized controlled trials.

Three studies found no difference in the overall incidence of oral mucositis between the control and intervention groups (31, 32, 35). One study found no difference between groups for the onset of mucositis and mucositis duration (34). However, four studies reported delayed onset of oral mucositis in patients supplemented with glutamine (28, 30, 33, 35). Furthermore, four studies reported a lower incidence of severe oral mucositis in the intervention group than in the control group (29, 30, 33, 35). The severity of oral mucositis was also reported to be significantly lower in the intervention group (28, 34).

Significantly later onset of dysphagia and less severe dysphagia (28) were observed in patients receiving glutamine compared to those who received placebo or no treatment. There were also reports of lower incidences of dermatitis (32, 37) and a shorter duration of dermatitis in the intervention group (37). However, another study found no difference in the development of dermatitis between the two groups (29). Significant weight loss was reported by two studies in the control group compared to the intervention group (28, 34), while two studies reported no difference between the two groups (30, 32). Tsujimoto et al. reported lower NRS scores in patients receiving glutamine supplementation (34).

Interestingly, Vidal-Casariego et al. evaluated the effects of early supplementation with glutamine against delayed supplementation with glutamine and no supplementation (36). There was a significant difference in the development of oral mucositis, whereby 75% of those with early supplementation, 94.7% of those with delayed supplementation, and 100% of those without supplementation developed oral mucositis. Less severe oral mucositis was also observed in patients who received early supplementation of glutamine. The same study also reported lower incidence and a smaller degree of weight loss in patients with early supplementation of glutamine, followed by delayed supplementation and no supplementation.

## Discussion

This systematic review summarizes recent evidence regarding the effect of immunonutrition in HNC patients undergoing radiotherapy and chemotherapy in nutrition status, functional status, and treatment-related toxicities.

Radiotherapy with or without chemotherapy is the most common mode of treatment for HNC as primary treatment or postoperative treatment (39). Even though current cancer treatment modalities are effective for tumor control; they are also associated with acute and late toxicities. Radiotherapy to the head and neck region is site-specific and localized, causing direct damage to cells in that area. This can damage nearby food consumption or digestion structures, such as taste buds and salivary glands. This will affect the early digestion process and taste changes, eventually leading to a loss of appetite and desire to consume food. Chemotherapy side effects can also lead to gastrointestinal symptoms, loss of appetite, and exacerbate radiotherapy side effects. Previous literature reported that HNC patients are at exceptionally high risk of malnutrition before initiation of radiotherapy and chemotherapy, and their nutrition status deteriorate further as the treatment progresses (6, 40–43).

Glutamine is a conditionally essential amino acid during metabolic stress. It is the primary fuel for the proliferation of lymphocytes, production of cytokines, and macrophage phagocytic and secretory activities (44). It is also the precursor for amino acids, proteins, nucleotides synthesis, and ammoniagenesis in the kidneys (45). Hence, glutamine may be beneficial in reducing mucosal damage during cancer treatment, including mucositis, stomatitis, pharyngitis, esophagitis and enteritis; and promote mucosal healing during and post-cancer treatment (46, 47). Arginine is involved in nucleotides, polyamines, nitric oxide, ornithine, citrulline and proline synthesis. Therefore, arginine has an essential role in the modulation of immune function, regulation of blood flow, angiogenesis and wound healing (46, 48). Omega-3 fatty acids, namely eicosapentaenoic acid (EPA) and docosahexaenoic acid (DHA), modulates the immune system by reducing the production of pro-inflammatory arachidonic acid (AA) and competes with AA for cyclooxygenase and lipoxygenase enzymes (49). Past literature suggests that omega-3 fatty acids may be associated with anticatabolic and antilipolytic activities (50).

The present systematic review found that overall, continuous supplementation with immunonutrient-enriched formulas may improve or maintain the nutrition status of HNC patients undergoing radiotherapy and chemotherapy. Six out of seven studies that implemented supplementation with immunonutrient-enriched formula during chemoradiation reported significant positive results in the intervention group compared to the control group receiving isocaloric, isonitrogenous nutrition supplementation or standard nutrition care. Maintenance or improvement in nutrition status is observed in subjects supplemented with formulas enriched with different combinations of omega-3 fatty acids with arginine and or glutamine. Even though nutrition status plays an essential role in the tolerance to treatment, treatment outcomes and survival, only 13 studies reported nutrition status as an outcome. It is also observed that the indicators used to measure nutritional status differ vastly among the studies. The most common indicator is weight or percentage of weight loss, while indicators like body composition or mid-arm circumference are less commonly used to measure nutrition status.

Changes in the functional status of HNC patients during cancer treatment is an important area in cancer management that have been of interest in the past two decades. Radiotherapy and chemotherapy treatment-related side effects such as oral pain, swallowing difficulty and nausea, can impair patients' quality of life significantly. However, the present systematic review only identified three studies that reported changes in the functional status of patients as an outcome of supplementation with immunonutrition. Two out of three studies reported improved functional status (Karnofsky Index scores) in the intervention group receiving immunonutrient-enriched formulas.

Studies that implemented supplementation with glutamine mostly reported treatment-related toxicities as the primary outcome, namely oral mucositis. Mucositis is the most common treatment side effect that occurs in HNC patients undergoing radiotherapy. In the present systematic review, three studies reported that there was no difference between groups being observed in terms of overall incidence (all grades) of oral mucositis. However, delayed onset of oral mucositis, less severe oral mucositis and lower incidence of severe oral mucositis was reported in eight other studies. Nutrition status was reported as a secondary outcome in seven studies. However, only three studies found significant positive results (less weight loss than the control group), while another two studies did not find any significant difference between the control and intervention groups. Even though glutamine was given in a modular supplementation, there were still positive outcomes in the nutrition status. This may be due to less severe oral mucositis in the intervention group, allowing for adequate intake of regular diet and oral nutrition supplement.

The present systematic review has several limitations. Due to the broad inclusion criteria, this systematic review included varied study designs, supplementation regimes and duration, and outcome measurements. The variability is high among studies that have been conducted in terms of timing and duration of supplementation, type of formula or combinations of immunonutrients, and dosage of immunonutrients or immunonutrient-enriched nutrition formula. Some studies included other primary cancer sites besides HNC, such as oesophageal, cervical and lung. Hence the outcomes were also inconsistent between studies. There were also very few RCTs with a large sample size. Furthermore, methodological quality and risk of bias were also of varying degrees, making it difficult to perform a more robust analysis or draw conclusions from the limited evidence available.

Even though our findings may not be conclusive, the positive effects of immunonutrition in HNC patients, whether in immunonutrient-enriched formula, or supplementation of single immunonutrient, or combination of immunonutrients; is still worth being investigated in future studies. Based on the systematic review findings, future studies should focus on well-designed, randomized controlled trials to investigate the effects of different dosages and combinations of immunonutrients in nutritional and functional status. Finally, future trials should also be more progressive, looking into the impact of timing and duration of immunonutrition, including supplementation prior to cancer treatment and continuation of nutrition supplementation post-treatment, which may further optimize the nutrition status of HNC patients and lead to better treatment outcomes.

## Conclusion

In conclusion, the present review found that supplementation with immunonutrient-enriched formulas in HNC patients during radiotherapy and chemotherapy may improve or maintain nutrition status. Supplementation with glutamine during HNC radiotherapy and chemotherapy may delay the onset of oral mucositis and reduce the incidence of severe oral mucositis. However, these findings are not conclusive, given the studies heterogeneity. Therefore, further investigations are encouraged in the future, focusing on the timing, dosage and duration of immunonutrition required for nutrition optimisation.

## Data Availability Statement

The original contributions presented in the study are included in the article/Supplementary Material, further inquiries can be directed to the corresponding author.

## Author Contributions

ST and HM contributed equally to the conception and design of the manuscript amd critical appraised the data selected. ST conducted the data collection and analysis and drafted the manuscript. All authors critically revised the manuscript, agree to be accountable for all aspects of work ensuring the integrity and accuracy of the manuscript, and read and approved the final manuscript. All authors contributed to the article and approved the submitted version.

## Funding

This review was funded by the University of Malaya Research Management Institute (RMF0002-2020). The sponsors had no role in the preparation of this manuscript. They did not take part in the design of the study, analysis and interpretation of data.

## Conflict of Interest

The authors declare that the research was conducted in the absence of any commercial or financial relationships that could be construed as a potential conflict of interest.

## Publisher's Note

All claims expressed in this article are solely those of the authors and do not necessarily represent those of their affiliated organizations, or those of the publisher, the editors and the reviewers. Any product that may be evaluated in this article, or claim that may be made by its manufacturer, is not guaranteed or endorsed by the publisher.

## Supplementary Material

Supplementary Material 1 Search Strategy for the Systematic Review.
