# Supplementary material for: Effects of Immunonutrition in Head and Neck Cancer Patients Undergoing Cancer Treatment – A Systematic Review
Source: Front Nutr. 2022 Feb 25;9:821924. doi: 10.3389/fnut.2022.821924 (PMC8961436; doi:10.3389/fnut.2022.821924)
Supplement: Supplementary Material 1 — Search Strategy for the Systematic Review. [file Table_1.DOCX]

**Search strategy for the systematic review**

| **Concept 1** |  | **Concept 2** |  | **Concept 3** |
| --- | --- | --- | --- | --- |
| Immunonutrition  OR  Immune enhancing nutrition  OR  Immune modulating nutrition  OR  Immunomodulatory nutrition supplement  OR  Glutamine  OR  L-glutamine  OR  Arginine  OR  L-arginine  OR  Omega  3 fatty acid  OR  Fish oil  OR  n-3 fatty acid  OR  Polyunsaturated fatty acid  OR  Eicosapentaenoic acid  OR  Docosahexaenoic acid  ­ | AND | Head and neck cancer  OR  Head and neck neoplasms  OR  Tongue cancer  OR  Mouth cancer  OR  Salivary gland cancer  OR  Tonsil cancer  OR  Oropharynx cancer  OR  Nasopharynx cancer  OR  Hypopharynx cancer  OR  Larynx cancer | AND | Chemotherapy  OR  Radiotherapy  OR  Chemoradiotherapy  OR  Chemoradiation therapy  OR  Concurrent chemotherapy and radiotherapy  OR  Cancer treatment |
